# Potential therapeutic targets for COVID-19 complicated with pulmonary hypertension: a bioinformatics and early validation study

**DOI:** 10.1038/s41598-024-60113-7

**Published:** 2024-04-23

**Authors:** Qingbin Hou, Jinping Jiang, Kun Na, Xiaolin Zhang, Dan Liu, Quanmin Jing, Chenghui Yan, Yaling Han

**Affiliations:** 1 State Key Laboratory of Frigid Zone Cardiovascular Disease, Cardiovascular Research Institute and Department of Cardiology, General Hospital of Northern Theater Command, Shenyang, China; 2 Department of Cardiology, Shengjing Hospital Affiliated to China Medical University, Shenyang, China

**Keywords:** COVID-19, Pulmonary hypertension, Differentially expressed genes, Machine algorithms, Core gene, Immune invasion, Molecular docking, Biocatalysis, Biochemistry, Biological techniques, Biophysics, Biotechnology, Biomarkers, Endocrinology, Health care, Medical research, Risk factors

## Abstract

Coronavirus disease (COVID-19) and pulmonary hypertension (PH) are closely correlated. However, the mechanism is still poorly understood. In this article, we analyzed the molecular action network driving the emergence of this event. Two datasets (GSE113439 and GSE147507) from the GEO database were used for the identification of differentially expressed genes (DEGs).Common DEGs were selected by VennDiagram and their enrichment in biological pathways was analyzed. Candidate gene biomarkers were selected using three different machine-learning algorithms (SVM-RFE, LASSO, RF).The diagnostic efficacy of these foundational genes was validated using independent datasets. Eventually, we validated molecular docking and medication prediction. We found 62 common DEGs, including several ones that could be enriched for Immune Response and Inflammation. Two DEGs (SELE and CCL20) could be identified by machine-learning algorithms. They performed well in diagnostic tests on independent datasets. In particular, we observed an upregulation of functions associated with the adaptive immune response, the leukocyte-lymphocyte-driven immunological response, and the proinflammatory response. Moreover, by ssGSEA, natural killer T cells, activated dendritic cells, activated CD4 T cells, neutrophils, and plasmacytoid dendritic cells were correlated with COVID-19 and PH, with SELE and CCL20 showing the strongest correlation with dendritic cells. Potential therapeutic compounds like FENRETI-NIDE, AFLATOXIN B1 and 1-nitropyrene were predicted. Further molecular docking and molecular dynamics simulations showed that 1-nitropyrene had the most stable binding with SELE and CCL20.The findings indicated that SELE and CCL20 were identified as novel diagnostic biomarkers for COVID-19 complicated with PH, and the target of these two key genes, FENRETI-NIDE and 1-nitropyrene, was predicted to be a potential therapeutic target, thus providing new insights into the prediction and treatment of COVID-19 complicated with PH in clinical practice.

## Introduction

The Severe Acute Respiratory Syndrome Coronavirus 2 (SARS-CoV-2) responsible for the coronavirus disease (COVID-19) has spread over the world and was proclaimed a pandemic on 11 March 2020, posing a major danger to public health all over the world^1–3^. Multiple mutant strains of SARS-CoV-2 have emerged during the past two years, boosting the virus's potential to infect people or elude vaccination protection. COVID-19 will continue to be a worldwide burden on healthcare and economies for as long as people are infected with it. In the post-pandemic period, we should also be concerned with the long-term effects of COVID-19 and its treatment^4–6^.

Long-term effects of COVID-19 can be observed in the respiratory and cardiovascular systems, but the virus also affects the neurological system, bones, and endocrine glands. Important COVID-19 consequences include acute respiratory distress syndrome (ARDS), venous thromboembolism, pulmonary hypertension, and acute cardiac injury^5,6^.

Pulmonary hypertension has been reported to complicate the course of illness for 13.4% and 21% of patients with a novel, and severe novel coronavirus infections, respectively. Pulmonary hypertension is a serious complication of new coronavirus infection, increasing the likelihood of requiring intensive care unit care, mechanical ventilation, extracorporeal membrane oxygenation (ECMO), and even death. Therefore, detecting high pulmonary artery pressure in SARS-CoV-2 patients early might enhance the long-term prognosis of patients and minimize the hospitalization rate and death owing to such complications^7–10^.The processes of immunological dysfunction, endothelial dysfunction, vascular leakage, and thrombotic microangiopathy that are comparable to those that cause pulmonary vascular disease may be responsible for the effects of SARS-CoV-2 on pulmonary hemodynamics, as revealed in previous studies. On the other hand, reports of the study mechanism's depth and specificity are uncommon^7–11^. This investigation aims to learn more about the connection between COVID-19 and pulmonary hypertension by examining the pathogenic molecules, pathological processes, and potential therapeutic targets involved.

**Figure 1:**
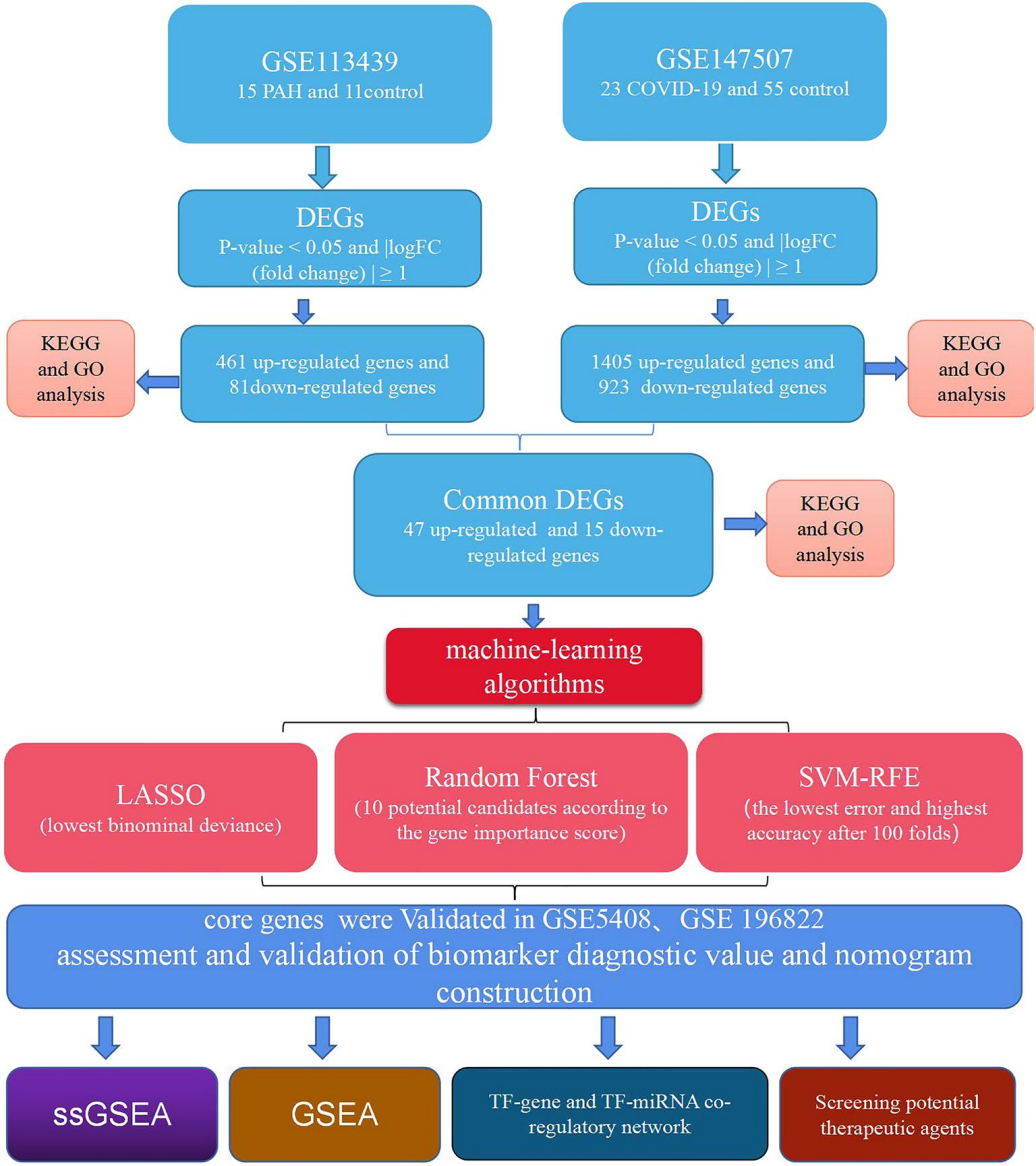
Research design flow chart.

Here, we ran a functional enrichment analysis on the GEO database to identify common differentially expressed genes (C-DEGs) across the COVID-19 and PH datasets. The validation queue confirmed the results of a screening of the key genes using three machine algorithms: LASSO, RF, and SVM-RFE-based. Gene set enrichment analysis (GSEA) was used to examine the role of prioritized core genes. We next mapped out the regulatory networks including these DEGs, including TF-gene connections and TF-microRNA co-regulation. Drug-protein interaction networks, molecular docking simulations and molecular dynamics simulations were employed to screen for possible therapeutic medicines. Our findings are expected to offer a novel approach to elucidating the genetic connection between the aforementioned disorders. Figure 1 depicts our research protocol.

## Results

### COVID- and PH-based DEGs screening

**Figure 2:**
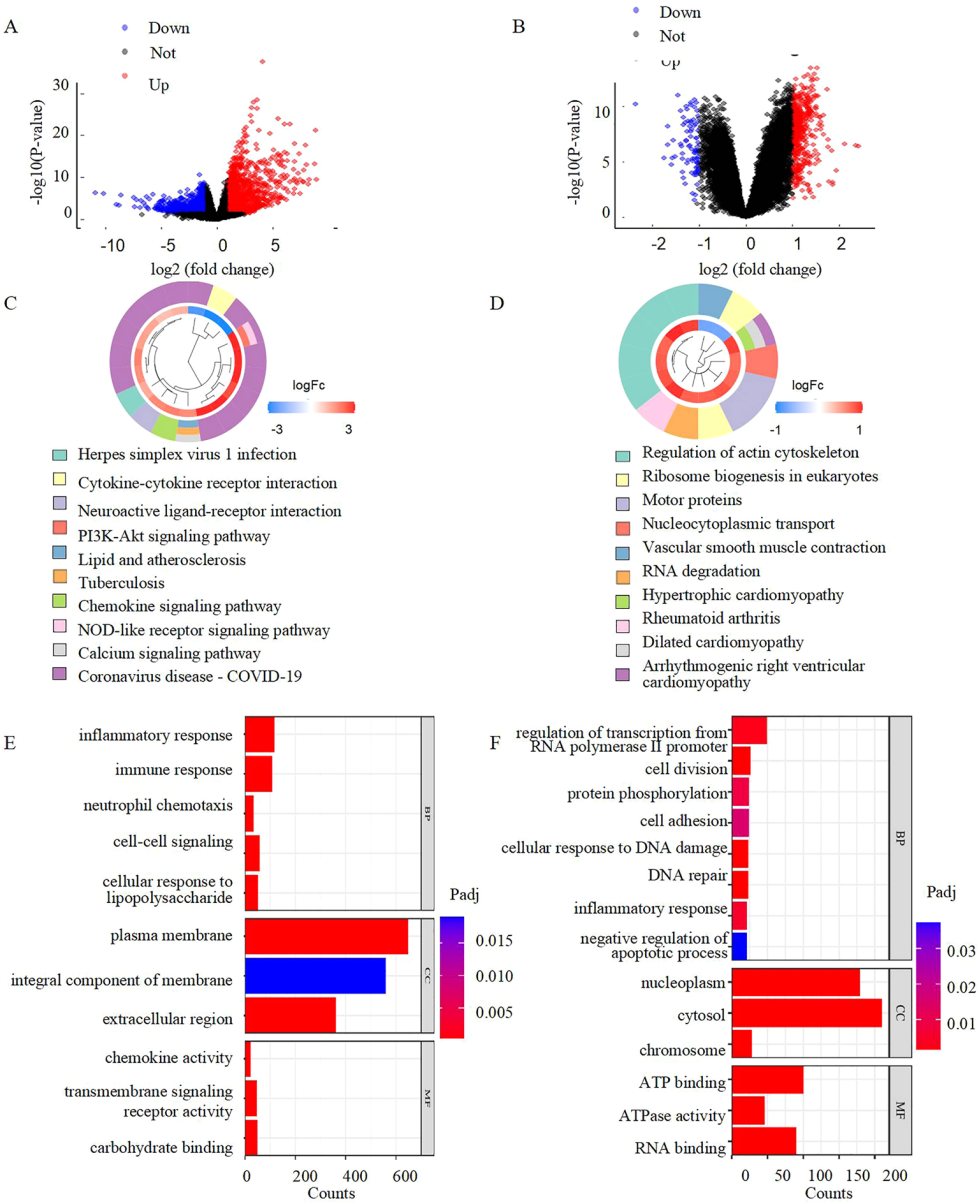
Screening DEGs for COVID-19 and PH respectively. (**A**) Volcano plot between COVID-19 patients and healthy controls.1405 up genes (red) and 923 down genes (blue). (**B**) Volcano plot between PH patients and healthy controls. 461 up genes (red) and 81 down genes (blue). (**C**,**D**)The KEGG analysis of COVID-19 DEGs, PH DEGs. (**E**,**F**) The GO analysis of COVID-19 DEGs、PH DEGs. *COVID-19* Coronavirus disease, *PH* pulmonary hypertention, *DEG* Differentially expressed genes, *KEGG* Kyoto Encyclopedia of Genes and Genomes, *GO* Gene Ontology.

There were 1405 differentially expressed genes (Fig. 2A) and 923 non-differentially expressed genes (Fig. 2B) between COVID-19 patients and controls in the GSE147507 dataset. As can be shown in Fig. 2B, 461 highly-expressed and 81 scarcely-expressed genes could be observed in the GSE113439 dataset distinguishing PH patients from healthy controls. Using the KEGG, we found that the PH dataset was enriched for the process of regulating the actin cytoskeleton (Padj = 0.031), ribosome biogenesis in eukaryotes (Padj = 0.0073), RNA degradation (Padj = 0.0072), nucleocytoplasmic transport (Padj = 0.022), contraction of vascular smooth muscle (Padj = 0.038), and motor proteins (Padj = 0.022). There was hypertrophic cardiomyopathy (Padj = 0.014), spondyloarthritis (Padj = 0.017), and right ventricular cardiomyopathy due to arrhythmia (Padj = 0.022) (Fig. 2C). The COVID-19 dataset, on the other hand, was enriched for Herpes simplex virus 1 infection (Padj = 0.000053), interaction between cytokines and their receptors (Padj = 0.00000002), neuroactive ligand-receptor interaction (Padj = 0.00086), PI3K-Akt signaling pathway (Padj = 0.016), lipid and atherosclerotic disease (Padj = 0.000044), tuberculosis (Padj = 0.00000083), chemokine signaling pathway (Padj = 0.0000051), NOD-like receptor signaling pathway (Padj = 0.000013), and COVID-19 (Padj = 0.025) (Fig. 2D). Moreover, analyzing genes using GO terms: enrichments of DEGs were seen in the COVID dataset in reaction to inflammatory response (Padj = 7.20 × 10^–26^), immune response (Padj = 8.40 × 10^–16^), neutrophil chemotaxis (Padj = 4.00 × 10^–14^), cell–cell signaling (Padj = 8.30 × 10^–12^), and cellular response to lipopolysaccharide (Padj = 1.30 × 10^–11^) (Fig. 2E). RNA polymerase II promote (Padj = 2.30 × 10^–03^), cell division (Padj = 1.30 × 10^–05^), protein phosphorylation (Padj = 8.50 × 10^–03^), cell adhesion(Padj = 0.015), cellular response to DNA damage (Padj = 3.30 × 10^–06^), DNA repair(Padj = 4.40 × 10^–06^), inflammatory response(Padj = 0.005), negative regulation of apoptotic process (Padj = 0.038) were enriched in the PH dataset, as shown in Fig. 2F.

### C-DEGs screening

**Figure 3:**
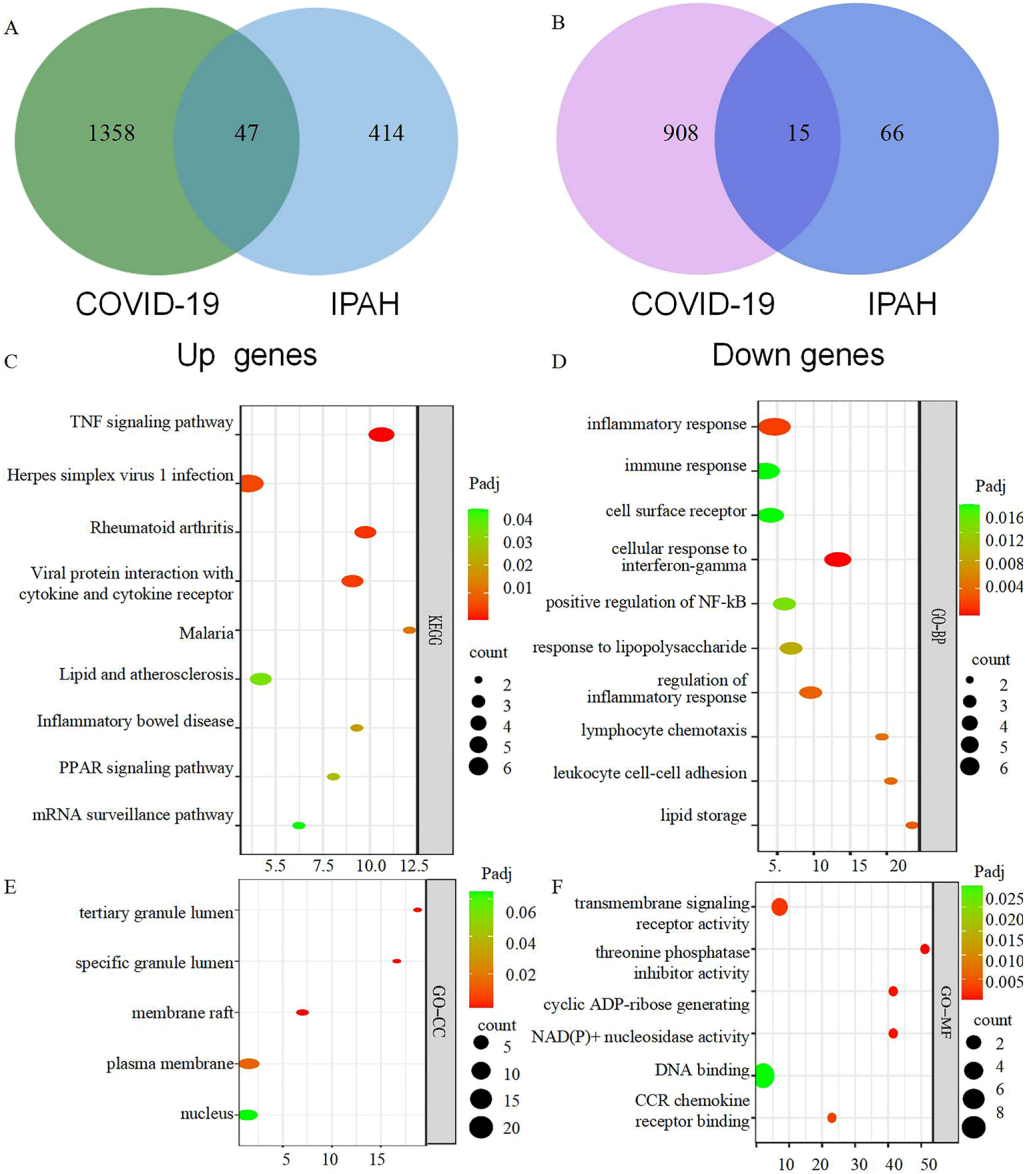
Identification and Functional enrichment analysis of common DEGs. (**A**) Venn diagram revealing 47 Up-regulated common DEGs in COVID-19 and PH. (**B**) Venn diagram revealing 17 Down-regulated common DEGs in COVID-19 and PH. (**C**) KEGG pathway analysis was performed on common DEGs. (**D**) GO-BP terms of common genes. (**E**) GO-CC terms of common genes. (**F**) GO-MF terms of common genes. *DEG* Differentially expressed genes. *COVID* coronavirus disease. *PH* pulmonary hypertention. *KEGG* Kyoto Encyclopedia of Genes and Genomes. *GO-BP, GO-CC, GO-MF* Gene Ontology terms in biological process, cellular component, and molecular function.

Using a Venn diagram to compare DEGs across the GSE147507 and GSE113439 datasets, we found 62 C-DEGs (Supplementary Table S1), 47 of which were significantly upregulated (Fig. 3A) and 15 downregulated (Fig. 3B).

### C-DEGs functional profiles evaluation

The C-DEGs exhibited enrichment in the TNF signaling pathway (Padj = 5.00 × 10^–4^), herpes simplex virus 1 infection (Padj = 0.0057), Rheumatoid arthritis (P adj = 0.4 × 10–3), viral protein interaction with cytokine and cytokine receptor (Padj = 0.0042), malaria (P adj = 0.012) lipid and atherosclerosis (Padj = 0.033), inflammatory bowel disease (Padj = 0.019), and PPAR signaling pathway (Padj = 0.025) (Fig. 3C, Supplementary Table S2).

The GO biological process (BP) enrichment investigation (Fig. 3D, Supplementary Table S3) revealed that C-DEGs were enriched for the following processes: inflammatory response (Padj = 0.0019), immune response (Padj = 0.017), cell surface receptor (Padj = 0.017), cellular response to interferon-gamma (Padj = 0.00023), positive regulation of NF-kB (Padj = 0.014), and response to lipopolysaccharide (Padj = 0.0094). Enrichment analyses for membrane raft (Padj = 0.00073), tertiary granule lumen (Padj = 0.00055), and specific granule lumen (Padj = 0.00078), transmembrane signaling receptor activity (Padj = 0.0025), threonine phosphatase inhibitor activity (Padj = 0.00068), and cyclic ADP-ribose generation (Padj = 0.001) were performed on C-DEGs (Fig. 3E,F). These findings suggest that immune inflammation plays a critical role in the etiology and progression of COVID-19 coupled with PH.

### Selection of candidate diagnostic biomarkers using machine learning

**Figure 4:**
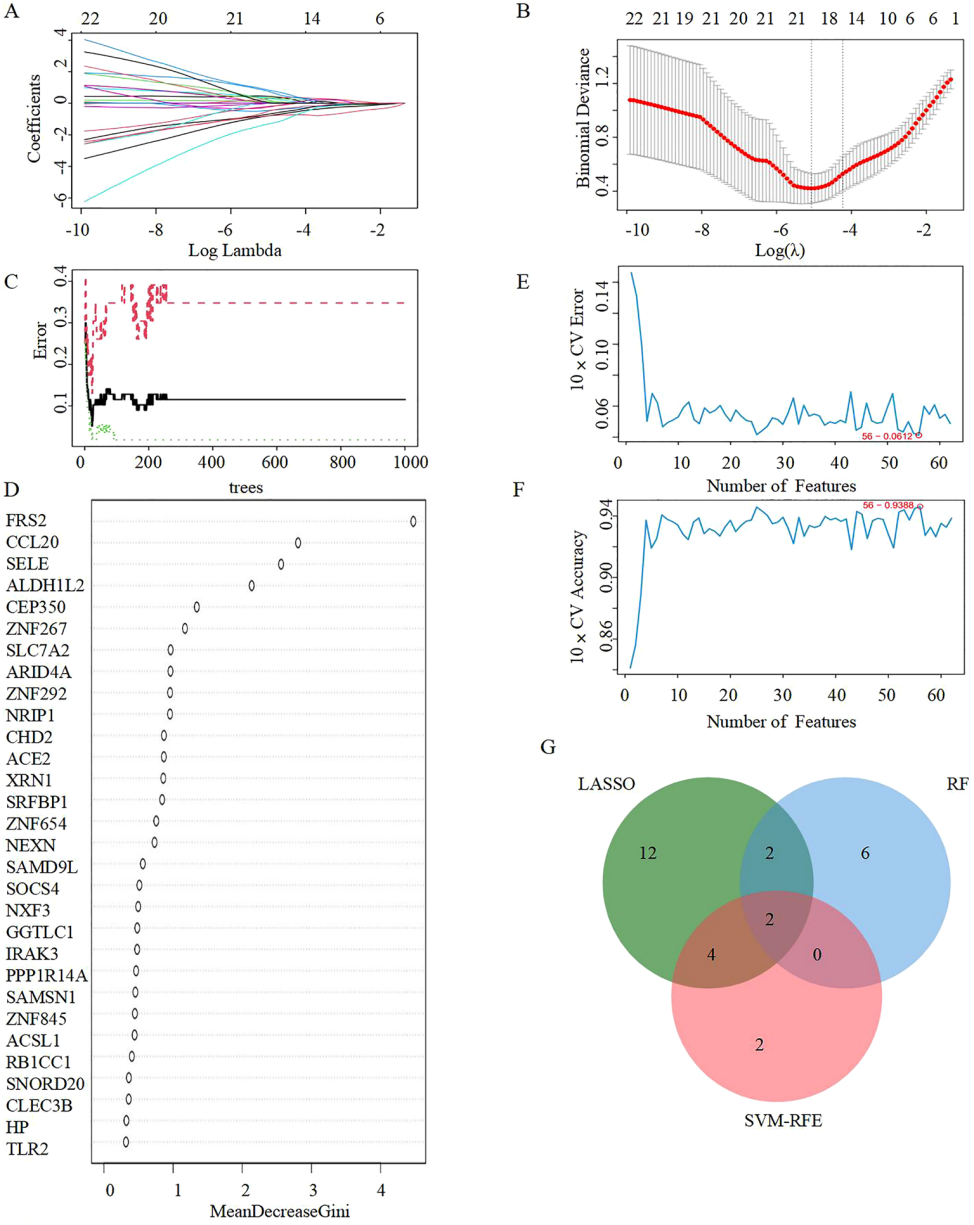
Selection of candidate diagnostic biomarkers of COVID-19 progression with machine learning approaches. (**A**,**B**) LASSO regression analysis was applied to screen diagnostic biomarkers. (**C**) The diagnostic error relating to control,COVID-19 and total groups was visualized from the random forest. (**D**) The column showing 30 DEGs ranked based on the importance score calculated from the random forest. (**E**,**F**) The number of DEGs with the lowest error and highest accuracy after 100 folds were considered the most suitable candidates via the SVM-RFE algorithm. (**G**) The intersection of 3 machine learning algorithms was obtained with a Venn diagram tool. *LASSO* least absolute shrinkage and selection operator, *SVM-RFE* support vector machine recursive feature elimination, *DEGs* Differentially expressed genes.

Among these 62DEGs (Supplementary Table S4), LASSO regression analysis identified 20 genes with the lowest binominal deviation (Fig. 4A,B). The random forest approach was used to identify 10 candidates after the DEGs were ranked by gene significance score (Fig. 4C,D). For PH progression in COVID19, the SVM-RFE method identified 8 genes with the lowest error and best accuracy after 100 folds (Fig. 4E,F). Finally, a Venn diagram was used to show how SELE and CCL20 (Fig. 4G) were shown to be DEGs when both methods were used together.

### Assessment and validation of biomarker diagnostic value and nomogram construction

**Figure 5:**
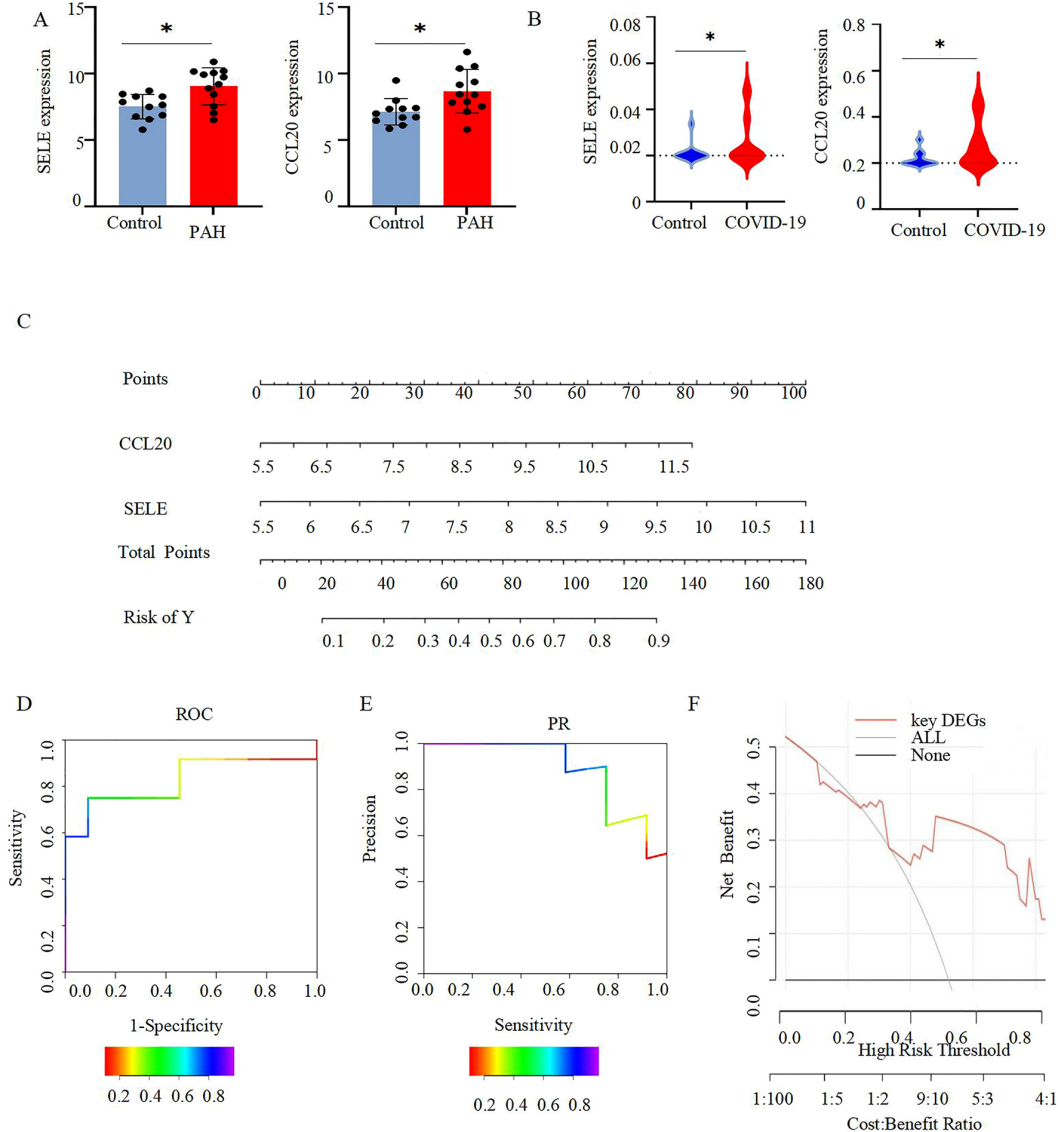
Nomogram construction and diagnostic performance validation. (**A**) SELE, CCL20 were validated in GSE53408.*P < 0.05. (**B**) SELE, CCL20 were validated in GSE196822.*P < 0.05. (**C**).The nomogram was established based on the 2 selected candidate biomarkers. Each DEG corresponds to a score. (**D**) The ROC curve of the nomogram. (**E**) PR analysis curve (**F**) decision curve analysis (DCA) curve.

The GSE53408, GSE196822 were used as validation dataset. There was a statistically significant difference in the expression of SELE and CCL20 between the pulmonary hypertension and control groups and between COVID-19 and control groups (Fig. 5A,B). After several iterations of selection, SELE and CCL20 were chosen to form a nomogram (Fig. 5C). Each gene's level of expression was given a numerical value in the nomogram. Finally, the sum score was applied to predict the incidence of PH progression in COVID-19 patients (Fig. 5D). Nomogram performed exceptionally well in predicting PH development in COVID19, with an AUC of 0.826 (95% CI 0.637–1.000; Fig. 5D). Moreover, precision recall (PR) and decision curve analysis (DCA) for the nomogram was also performed, showing that the nomogram model may be beneficial for the diagnosis of COVID-19 complicated with PH (Fig. 5E,F).

### GSEA of Hub genes

**Figure 6:**
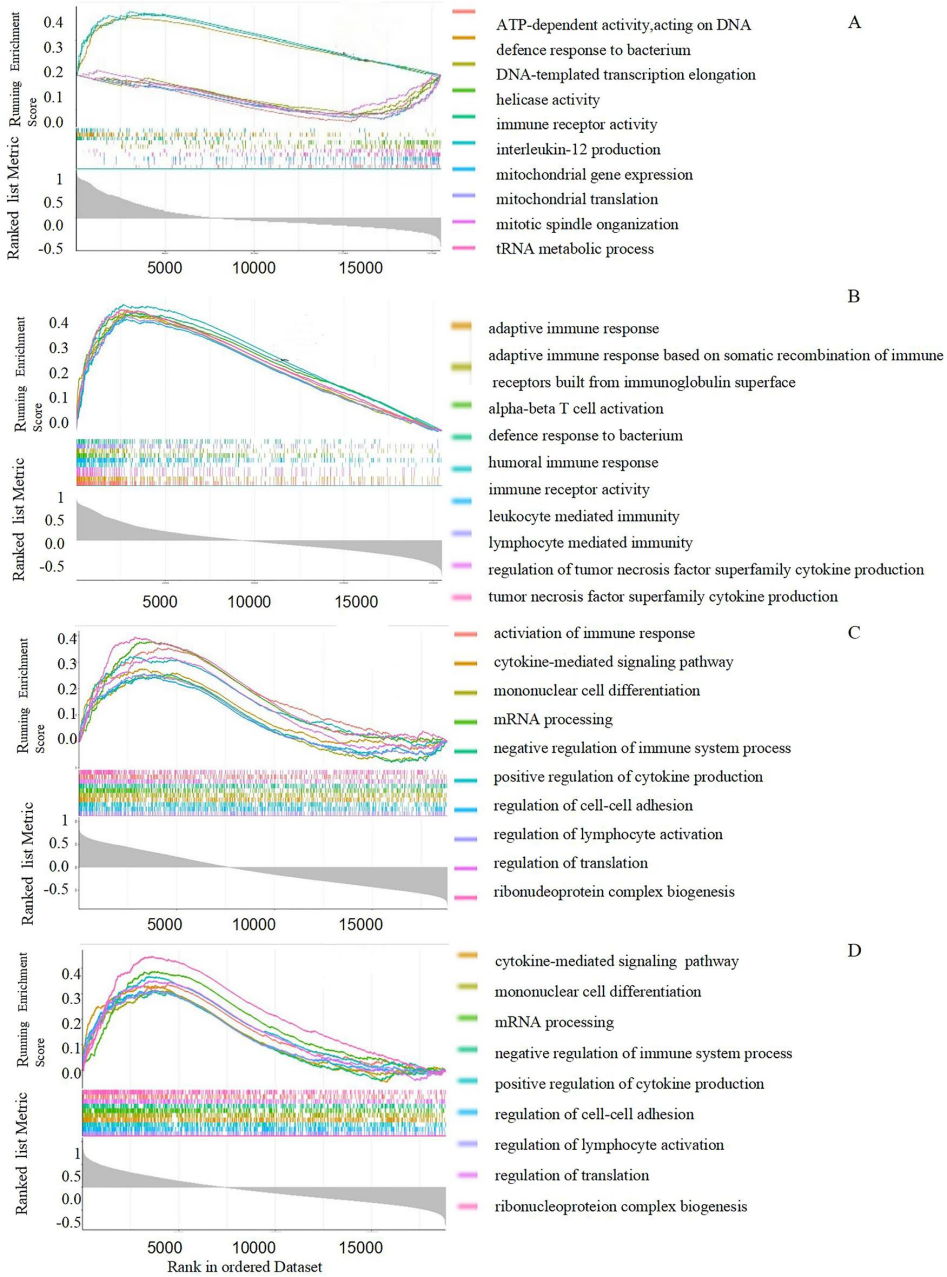
Gene set enrichment analysis. (**A**,**B**) A merged enrichment plot of SELE, CCL20 in GSE 147507 cohort. (**C**,**D**) A merged enrichment plot of SELE, CCL20 in GSE113439 cohort.

We used GSEA to determine the probable physiological functions of the two hub genes between COVID-19 and PH. We found that increased SELE, CCL20 expressions in GSE113439 (Fig. 6A,B) and GSE147507 (Fig. 6C,D) were strongly associated with activated immune responses such as adaptive immune response around blood vessels. The increase of proinflammatory factor increased the expression of cytokines and chemokines, contributing to excessive contraction and proliferation of blood vessel cells.

### Immune invasion and core genes correlation

**Figure 7:**
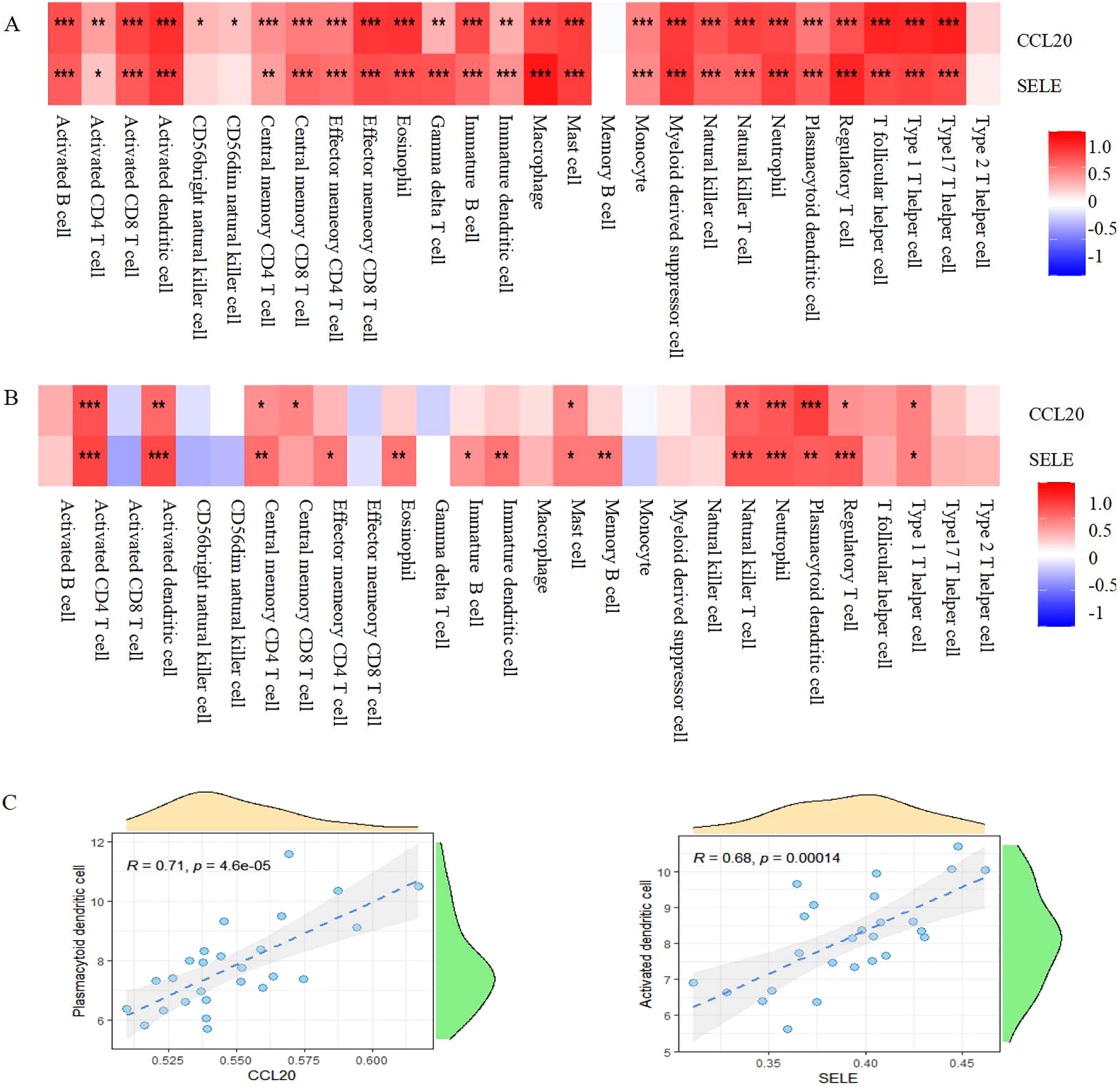
Association between the hub genes and immune infiltration. (**A**) In GSE147507cohort, CCL20, SELE were shown to positively correlate with most cell types. Except for CD56dim natural killer cells, CD56bright natural killer cells, memory B cells, and Type 2 T helper cells. (B) In GSE113439 cohort, CCL20, SELE were shown to positively correlate with many cell types. Including: type 1 T helper cell, regulatory T cell, plasmacytoid dendritic cell, neutrophil, natural killer T cell, mast cell, central memory CD4 T cell, activated dendritic cell, and activated CD4 T cell. Red: positive correlation; Blue: negative correlation. (**C**) According to a Pearson correlation study, plasmacytoid dendritic cells had the highest level of association with CCL20,while Activated dendritic cells had the highest level of linkage with SELE.

We used ssGSEA for examining the possible relationships between the two discovered hub genes and 28 immune cells. SELE、CCL20 were shown to be directly related with various immune cell types in the GSE 147507dataset (Fig. 7A), except for CD56dim natural killer cells, CD56bright natural killer cells, memory B cells, and Type 2 T helper cells. These two found hub genes in GSE113439 were linked to the following cell types and subsets: type 1 T helper cell, regulatory T cell, plasmacytoid dendritic cell, neutrophil, natural killer T cell, mast cell, central memory CD4 T cell, activated dendritic cell, and activated CD4 T cell (Fig. 7B).

When we combined the ssGSEA findings from the aforementioned datasets, we found that COVID-19 and PH were linked to the activation of CD4 T cells, activated dendritic cells, natural killer T cells, neutrophils, and plasmacytoid dendritic cells. According to a Pearson correlation study, plasmacytoid dendritic cells had the highest level of association with CCL20, while Activated dendritic cells had the highest level of linkage with SELE (Fig. 7C).

### Co-modulatory axis TF-miRNA and TF-gene associations

**Figure 8:**
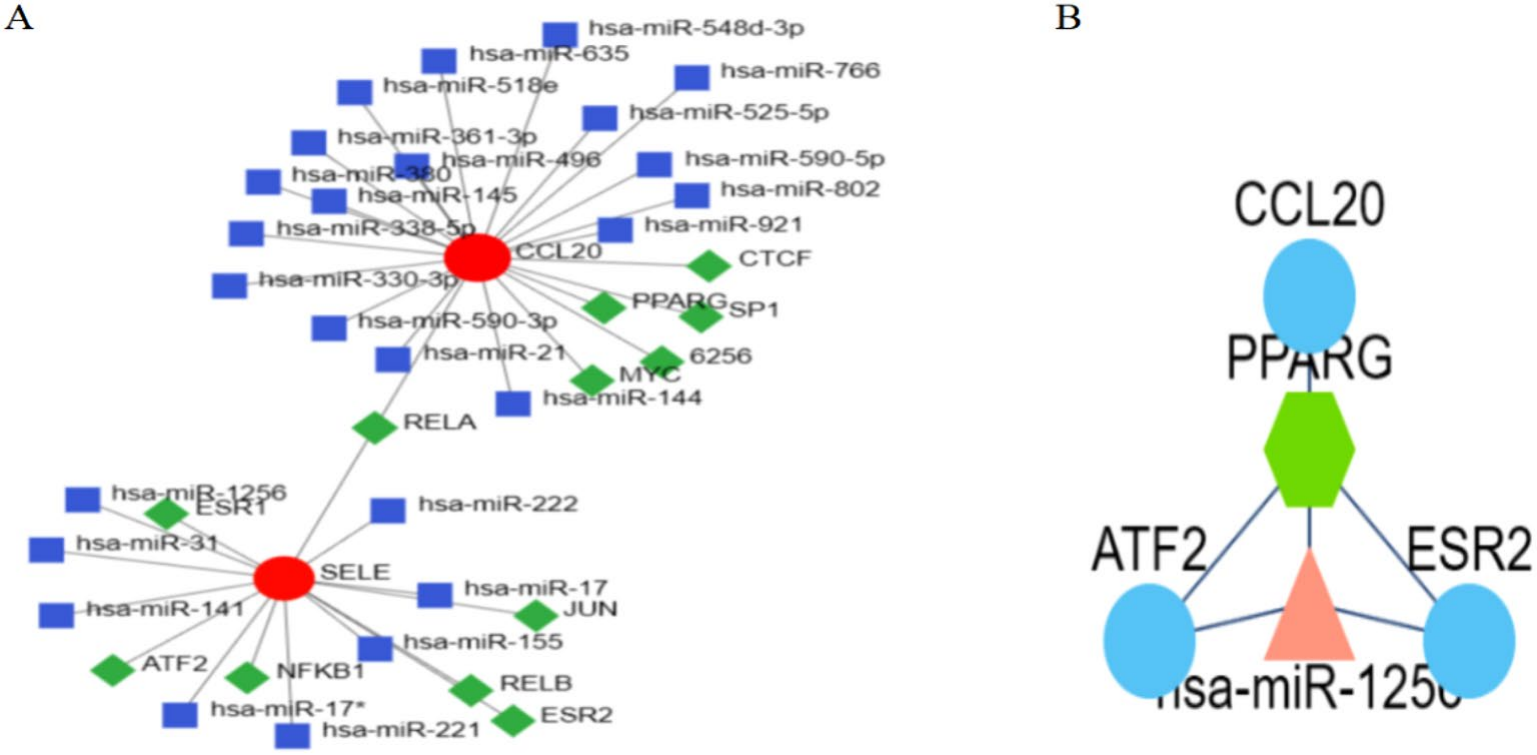
Network for TF-gene and TF-miRNA interaction with Common DEGs. (**A**) We predicted 25 miRNAs and 11 TF-genes interacting with SELE and CCL20 through the Network analyst website. Red nodes: hub genes; blue nodes: miRNA. Green nodes:TF-genes. (**B**) We identified that among CCL20, miR-1256 and PPARG may be a novel regulatory module in COVID-19 complicated with pulmonary hypertension through the FFL tool.

After that, the Network analyst was utilized (Fig. 8A). There was a total of 19 nodes and 18 edges in the constructed network. There was a strong correlation among hub genes and TFs, and the TFs affected more than one hub gene in the network. To assess the connection between miRNAs + TFs and hub genes, we built the TF miRNA co-modulatory axis. There were 39 nodes in our network, 38 edges, and 25 miRNA-based interactions that involved hub genes, by which the overall expression of hub genes could be modulated. In our research, SELE and CCL20 have been identified as potential diagnostic biomarkers for COVID-19 in conjunction with pulmonary hypertension (PH). To further validate the roles of these genes in the disease, we propose the use of the FFLtool for analyzing transcription factor-gene and transcription factor-miRNA networks. FFLtool is a web server designed for the analysis of feed-forward loops (FFLs), providing deep insights into the interactions between transcription factors, miRNAs, and genes. By incorporating our key genes, transcription factors, miRNAs into this analysis. As a result, the FFL containing TF CCL20, miRNA miR-1256 and target gene PPARG appeared at the top (Fig. 8B,Supplementary Fig. 1). This FFL among CCL20, miR-1256 and PPARG may be a novel regulatory module in COVID-19 complicated with pulmonary hypertension.

### Targeted chemical interaction and candidate drugs identification in COVID-19 and PH

Glutathione, Simvastatin, niacin, Fenretinide, 1-nitropyrene, *N*-acetyl-l-cysteine, nickel chloride, silica, vincristine, hydrogen peroxide, and aflatoxin B1 were among the 10 medications under consideration (Supplementary Table S5). These potential medications interacted with shared DEGs, suggesting they may be used to treat both disorders.

**Figure 9:**
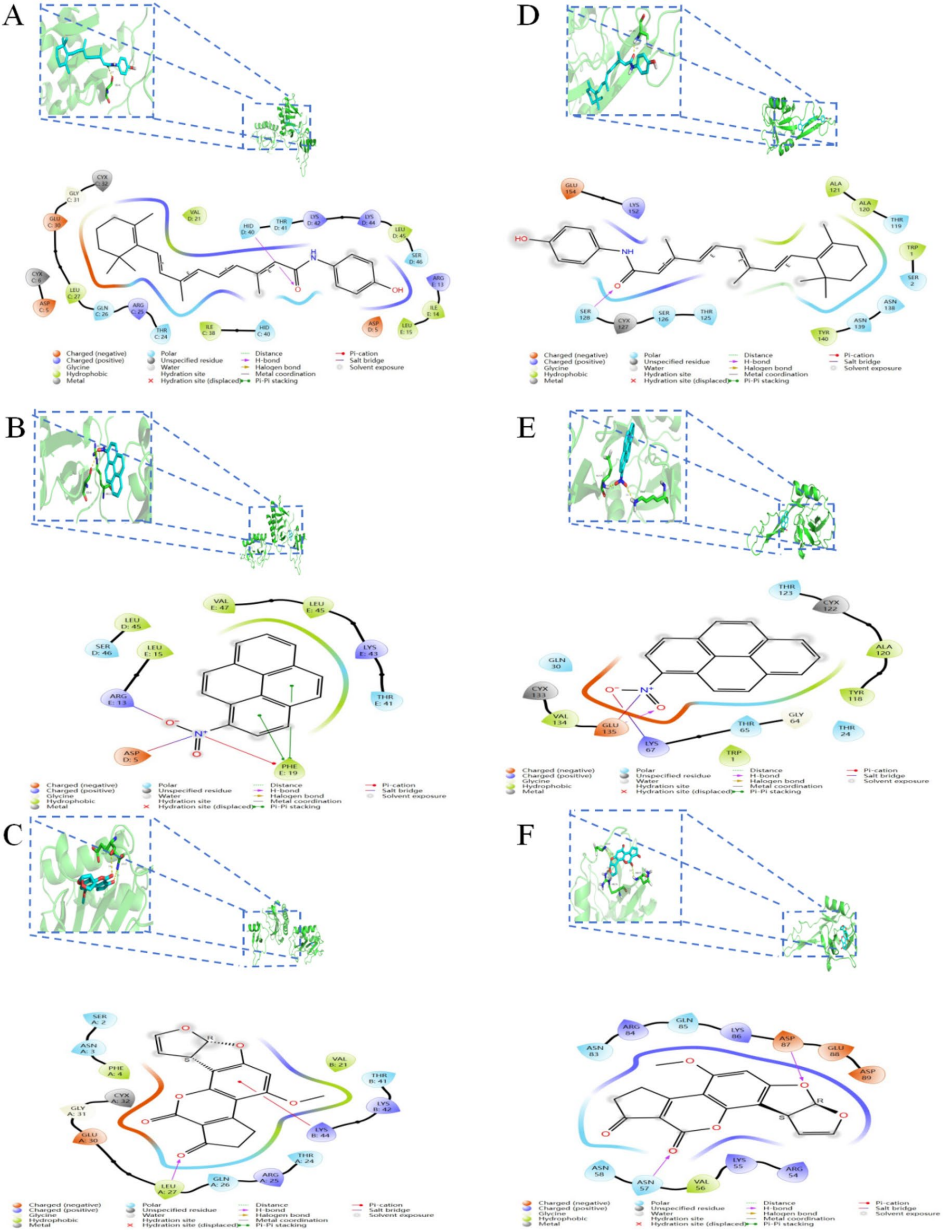
3D and 2D Molecular docking patterns for (**A**) FENRETINIDE, (**B**) 1-NITROPYRENE, (**C**) AFLATOXIN B1 with CCL20 respectively. (**D**) FENRETINIDE, (**E**) 1-NITROPYRENE, (**F**) AFLATOXIN B1 with SELE respectively.

In addition, molecular docking was utilized to foretell the drug-hub gene binding mechanisms (Supplementary Table S6). Figure 9 displays the outcomes of the molecular docking analysis. It was discovered that the binding sites of FENRETINIDE, 1-NITROPYRENE, and AFLATOXIN B1 to the two target proteins have lower stabilization energies. As a result, these three medications under consideration may one day be used to treat both COVID-19 and PH (Fig. 9). Subsequently, we also performed drug predictions for these two key genes in the CTD database, we found that Lipopolysaccharides, bisphenol A, Acetaminophen, Benzo(a)pyrene, Silicon Dioxide, Tetrachlorodibenzodioxin, titanium dioxide, 1-nitropyrene, 2,2′,4,4′-tetrabromodiphenyl ether, 2-anisidine (Supplementary Table S7).Combining Enrichr and CTD databases is not difficult to find 1-NITROPYRENE can be predicted in both databases. So we further performed molecular dynamics simulations.

### Molecular dynamics simulation of 1-nitropyrene-CCL20/SELE complex

Based on the molecular docking results, 100-ns MD simulations of the 1-nitropyrene-CCL20/SELE complex were performed to investigate the dynamic properties obtained by molecular docking. The final results included the Root Mean Square Deviation (RMSD), Root Mean Square Fluctuation (RMSF), Radius of Gyration (Rg), Solution Accessible Surface Area (SASA), and the changes in hydrogen bonds during the entire 100-ns simulation process.

**Figure 10:**
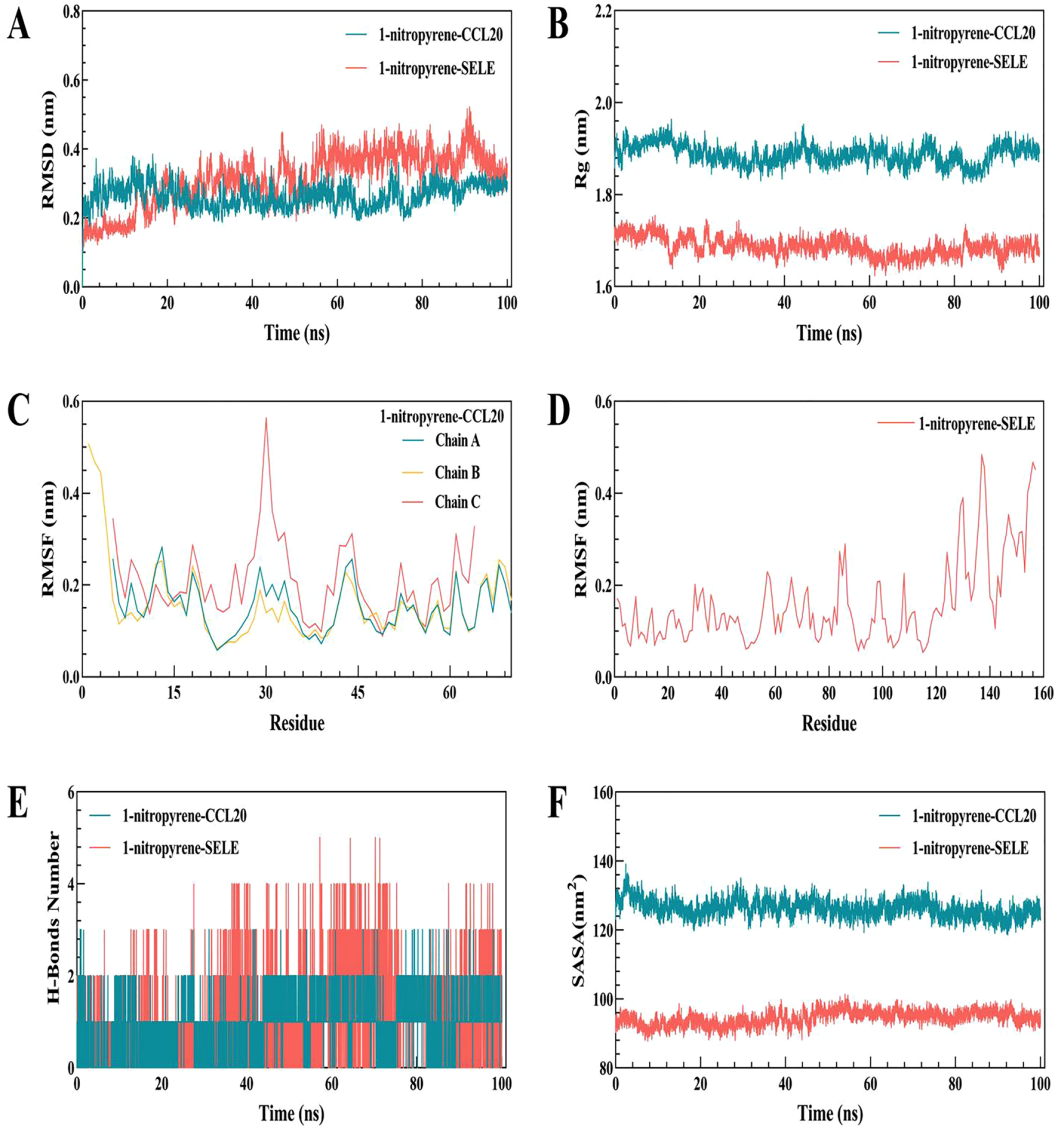
100 ns molecular dynamics simulation analysis of 1-nitropyrene-CCL20/SELE complex. (**A**) RMSD curve of 1-nitropyrene-CCL20/SELE complex, (**B**) Rg curve of 1-nitropyrene-CCL20/SELE complex, (**C**) RMSF curve of 1-nitropyrene-CCL20 complex, (**D**) RMSF curve of 1-nitropyrene-SELE complex, (**E**) hydrogen bond change curve of 1-nitropyrene-CCL20/SELE complex, and (**F**) SASA curve of 1-nitropyrene-CCL20/SELE complex.

The RMSD curves of the 1-nitropyrene-CCL20 complex gradually approached equilibrium after 28 ns, with an RMSD fluctuation of 0.25–0.3 nm. In addition, the RMSD curves of the 1-nitropyrene SELE complex all approached equilibrium at 60–88 ns, and the mean RMSD was less than 0.35 nm (Fig. 10A). The Rg curves of the 1-nitropyrene-CCL20 and 1-nitropyrene-SELE complexes remained stable during the entire process, with average Rg values of 1.88 nm and 1.68 nm, respectively (Fig. 10B). The low Rg values indicated that the protein remained compact during the simulation process of 100 ns. In the 1-nitropyrene-CCL20 complex, the RMSF of the C chain of CCL20 protein fluctuated significantly; this was especially noticeable in the region of amino acids 27–36 which, as can be seen from the protein structure, is located in the loop region of the C chain, resulting in greater flexibility during the 100-ns process. The RMSF values of the α-helix and β-fold in the C chain remained relatively low during the simulation process, while the RMSF values of the A and B chains were observed to change essentially simultaneously, indicating that small 1-nitropyrene molecule had little effect on the overall flexibility of the CCL20 protein. In the 1-nitropyrene-SELEcomplex, large RMSF values were observed in the 124–140 amino acid region which, according to the molecular docking, surrounds the binding pocket of the small molecule. Therefore, we speculate that the addition of 1-nitropyrene increased the flexibility of the amino acids at the binding site. The results show a large RMSF value (Fig. 10C,D). In the 100-ns simulation process, the number of hydrogen bonds in the 1-nitropyrene-CCL20/SELE complex was maintained at 2, while the maximum number of hydrogen bonds in the 1-nitropyrene-CCL20 complex was 3. The 1-nitropyrene-SELE complex could have up to five hydrogen bonds. This considerable number of hydrogen bonds helps to maintain the stability of the complex (Fig. 10E). The SASA curves of the 1-nitropyrene-CCL20 and 1-nitropyrene-SELE complexes remained stable throughout the process, and the mean SASA values were as follows: The low SASA values of 128 nm^2^ and 92 nm^2^ (Fig. 10F).

## Discussion

After the initial observation by Xie and colleagues^12^ that COVID-19 is associated with an increase in cardiovascular disorders, numerous clinical studies and meta-analyses have confirmed an augmented incidence of acute coronary syndromes, myocarditis, pericarditis, heart failure, and arrhythmias^13–20^ The consequences of severe COVID-19 include systemic hypoxia, acute respiratory distress, hyper-coagulation, sepsis, inflammation, metabolic stress, and cytokine storms, all of which may stress the cardiovascular system, eventually leading to blood pressure dysregulation^21–25^.

Pulmonary hypertension, as a serious cardiopulmonary complication of COVID-19,increasing the likelihood of requiring intensive care unit care, mechanical ventilation, extracorporeal membrane oxygenation (ECMO), and even death. Therefore, detecting high pulmonary artery pressure in SARS-CoV-2 patients early might enhance the long-term prognosis of patients and minimize the hospitalization rate and death owing to such complications^7–9,26^.But de Jong CMM^27^ found that chronic thromboembolic pulmonary hypertension is not a more common long-term complication after COVID-19-associated pulmonary embolism than after non-COVID-19-associated pulmonary embolism. Whether this phenomenon is due to large differences in pathogenesis, it is particularly important to study the pathogenesis of COVID-19 combined with pulmonary hypertension.

So, this study attempted to explore molecules associated with the pathogenesis of COVID combined with PH through a variety of bioinformatics methods, after which a comprehensive diagnostic pattern was established by scoring significant markers. The expression of each gene was quantified and scored, with higher scores associated with greater predictive potential. These predictive scores could be used for monitoring and early intervention in COVID-19 patients, especially those with PH. A further aim was the identification of specific drugs that could target key genes associated with COVID-19 complicated with PH, enhancing both the diagnosis and treatment of this condition.

Similar to the genetic causes of pulmonary arterial hypertension, COVID-19 severity may be affected by variations in the same genes. Many of the pathobiological hallmarks of pulmonary arterial hypertension are also present in COVID-19-induced pulmonary vasculopathy, including endodermatitis, vasculitis-medial hypertrophy, and smooth muscle cell proliferation. Especially endothelial dysfunction is a common feature of the clinical manifestations observed in COVID-19 patients. The SARS-CoV-2 coronavirus accesses host cells via the binding of its spike glycoprotein to angiotensin-converting enzyme 2(ACE2), sialic acid receptor, transmembrane serine protease 2 (TMPRSS2), and extracellular matrix metalloproteinase inducer (CD147); catepsin B and L also participate in virus entry. All of these factors are expressed in endothelial cells^28–31^. Overactivated platelets cause cytokine storms and thrombosis, and studies have shown that platelets that express pro-inflammatory molecules and that carry viral RNA are particularly likely to be highly active^32^.

Among the C-DEGs, we found 47 highly expressed genes and 15 lowly expressed genes when comparing COVID-19 and PH datasets. Using GO analysis, we found that C-DEGs were enriched for the following terms: inflammatory response, immunological response, cell surface receptor, cellular response to interferon-gamma, positive regulation of NF-kB, and reaction to lipopolysaccharide. TNF signaling route, Herpes simplex virus 1 infection, viral protein interaction with cytokine and cytokine receptor, malaria, lipid and atherosclerosis, PPAR signaling pathway, and PPAR signaling pathway were all enriched for among the C-DEGs in the KEGG enrichment analysis. These results strongly suggest that immune inflammation is a driving force in the emergence and progression of COVID-19 coupled with PH. Infection with SARS-CoV-2 may cause a cytokine storm leading to systemic inflammation and vascular endothelial cell damage. These changes may cause hypercoagulability and intravascular thrombosis, together with increasing pulmonary vascular resistance. Diffuse microangiopathy and microthrombosis caused by extensive impairment of vascular endothelial function may further aggravate the imbalance of the pulmonary ventilation/blood flow ratio, and increases in the pulmonary right-to-left shunt may further aggravate hypoxia, thus promoting pulmonary vascular contraction and remodeling. In addition, when the body is attacked by a virus, it can activate T cells and promote the overexpression of IFN-induced genes that could also lead to the apoptosis of endothelial cells and thus to PH. This has much in common with the results of LuisG's study^33^.

To further search for the core genes associated with COVID-19 combined with PH among the 62 identified DEGs, we used machine learning to choose potential diagnostic biomarkers. LASSO regression analysis identified 20 genes with the lowest binominal deviation. The random forest approach identified 10 candidates after ranking the DEGs according to significance, and the SVM-RFE method identified 8 genes with the lowest error and best accuracy after 100 fold changes. Using the intersection of the results of the three algorithms, SELE and CCL20 were identified as core genes. These two key genes were discovered to have statistically significant differences when compared to one another in the validation set. Next, we built a prediction model to make even more informed predictions on COVID-19 complicated with PH by assessment of the scores in the table in which higher scores represented greater likelihood of developing PH after COVID-19. By drawing the ROC, PR, and DCA curves, it was found that the prediction model was reliable.

One of the core genes identified was SELE. Activation of endothelial cells by cytokines results in the expression of a cell-surface glycoprotein called SELE, which facilitates the adherence of circulating monocytes and lymphocytes to endothelial cells^34^. The plasma marker for endothelial dysfunction or injury is soluble E-selectin (sE-selectin), which is secreted by damaged or dysfunctional endothelial cells^35,36^. IFN-gamma-R2 membrane transport to the Golgi and proper IFN-gamma-R assembly both need E-selectin involvement. The activation of the BTK kinase is triggered by the interaction of an E-selective protein, which in turn forms a functional IFN-gamma-R that can bind to another functional IFN-gamma-R, so generating an efficient innate response of macrophages to intracellular bacterial infection^37,38^. The importance of endothelial cells in the spread of SARS-CoV-2 is becoming more widely accepted. It is probable that E-Selectin's function in leukocyte chemotaxis during inflammation is the fundamental mechanism at work here. E-Selectin surface expression increases, which may facilitate the entry of leukocytes into the tissue and the initiation of inflammation to combat the infection^39,40^.DM Smadja et al. discovered that PH is linked to circulating endothelial cells, soluble E-selectin, and sVCAM, but not to endothelial progenitor cells, CD34(+)CD133(+) cells, or vascular endothelial growth factor (VEGF)^41^. Similarities between this and our study's results suggest that SELE may be a key pathogenic molecule of COVID 19 coupled PH.

CCL20, also known as macrophage inflammatory protein-3α or liver activation regulated chemokine, is another gene of central importance that we examined. CCL20 is a CC chemokine that specifically interacts to CCR6. In addition to recruiting immature dendritic cells, effector/memory T cells, and B cells, this chemokine also has an inflammatory role in maintaining homeostasis. It plays a crucial part in maintaining regular trafficking of immune cells and in kicking off T-cell-dependent inflammation. CCL20 regulates the right amount of inflammation by keeping a fine balance between offensive and defensive immunity^42–45^. It also recruits Th17 cells and regulatory T cells to inflammatory sites since CCR6 is present on these cell types. Patients with COVID-19 were found to have elevated levels of CCL20 in both bronchoalveolar lavage (BAL) fluid and plasma samples^46^. Pulmonary arterial hypertension in individuals with SSc is associated with elevated serum CCL20 levels^44^. However, there has only been a little amount of research done on CCL20 in COVID and PH patients.

Functional enrichment was primarily in adaptive immune response, leukocyte, and lymphocyte mediated the immune response, and proinflammatory response mediated by cytokines like IL-12 and TNF-a, which promoted the proliferation of pulmonary artery smooth muscle cells and induced vascular remodeling, as determined by GSEA of SELE and CCL20 in the data sets of COVID-19 and PH.

We used ssGSEA analysis to look at the relationship between SELE and CCL20 and 23 different types of immune cells in the COVID-19 and PH datasets, and we found that activated CD4 T cells, activated dendritic cells, natural killer T cells, neutrophils, and plasmacytoid dendritic cells were all linked to COVID-19 and PH. The highest Pearson correlation was reported between CCL20 and plasmacytoid dendritic cells, and the highest connection between SELE and activated dendritic cells. The immunological response of different cells may be regulated by SELE and CCL20, leading to an increase in the incidence of COVID-19 coupled PH. Lymphocytes, including NK cells, are activated and migrate to the lung during an acute coronavirus infection because of the accumulation of inflammatory mononuclear macrophages and neutrophils, which release cytokines and chemokines. When SELE and CCL20 levels drop, NK cell numbers and function decline. PH developed as a result of an increase in sensitivity to COVID-19 and modification of pulmonary artery walls caused by NK cell destruction. Chronic tissue inflammation can be caused by CD4 + T cell-mediated cellular immunity, which is a particular cellular immune response driven by CD4 + T cells^47–50^. There is an invasion of lymphocytes (mostly T cells) and mononuclear phagocytic cell lines, resulting in an exudative inflammation. The pathophysiology of COVID-19-complicated PH is also influenced by SELE and CCL20-mediated neutrophil proliferation. By secreting neutrophils extracellular traps, which in turn increase pulmonary artery endothelial cell damage and smooth muscle cell proliferation, neutrophils serve to both perpetuate and worsen inflammation.

SELE, CCL20 with dendritic cells exhibited the greatest correlation among the five cell types, according to a Pearson analysis. Dendritic cells were dramatically decreased in individuals with COVID-19 sequelae, according to research by Tomonari Sumi et al.^51^. About 7 months after SARS-CoV-2 infection, Perez-Gomez A et al^52^ discovered that dendritic cells dropped considerably in vivo. There are parallels between this conclusion and our own. There is a decrease in the number of mature myeloid DC and associated functional abnormalities in people with COVID-19 who also have PH. Therefore, DC cells are unable to initiate an immune response by encouraging primary T cell activation and proliferation in order to protect the body from virus-induced harm.

In addition to diagnosis, the identification of drugs targeting pathogenesis is also an important direction for us to explore. Current treatment of COVID-19 is primarily dependent on supportive care, together with the use of antiviral and immunomodulatory drugs. Given the distribution of the population living with comorbidities, specifically, the predominantly middle-aged and elderly demographic, poly pharmaceuticals and drug-drug interactions might be apparent. Unfortunately, the potential risk of drug-drug interactions is largely unknown since most studies on COVID-19 do not provide details on interactions between drugs used in the course of COVID-19 treatment and co-medications used for the management of other comorbidities in these patients^53^. Moreover, the use of some commonly used drugs in COVID patients may lead to an increased number of adverse pulmonary effects^54^. The current direction is to find suitable drugs that have a certain therapeutic effect and relatively few side effects. In this study, because of the significance of SELE and CCL20 in the pathogenesis of COVID-19 and PH, we chose them as drug prediction and molecular docking targets. The binding energy of these two molecules with FENRETINIDE, 1-NITROPYRENE, and AFLATOXIN B1 was the lowest of any of the eight anticipated medications, which was not expected. At present, there are few reports that 1-NITROPYRENE and AFLATOXIN B1 can improve the symptoms of COVID-19 and PH^55–62^.Also as a nitro compound, WenXia Feng et al. found that Inhaled nitric oxide treatment was beneficial in reducing and stabilizing the PASP and might also reduce the risk of right heart failure in COVID‐19 with pulmonary hypertension^63^. In this study, we found that whether in the enrichr database or the CTD database, by performing drug predictions on the key genes that COVID combines with PH, we found that we could predict that 1-NITROPYRENE was a potential therapeutic drug, and that through molecular docking and molecular dynamics simulations, we found that the combination of 1-NITROPYRENE and the two key genes was extremely stable. Therefore, it has great potential to be used as a drug to treat this complication. Because of its beneficial effects on glucose tolerance, lipid levels, and body fat percentage, the synthetic retinide derivative Fenretinide has been used for a variety of medical purposes, including cancer prevention and treatment, atherosclerosis improvement, and the amelioration of non-alcoholic fatty liver disease. Its capacity to reduce the production of inflammatory mediators and prevent macrophage polarization may be the primary mechanism at work here^63–65^. Fenretinide was found to inhibit the release of pro-inflammatory factors (IL-1β, MCP1, iNOS, and TNF-α). Fenretinide may inhibit NF-κB signaling by reducing the nuclear translocation of the protein via downregulation of IKKβ and IκBα phosphorylation^64^. In addition, delayed release of IFN-I is well known in SARS-CoV infection as a mechanism that slows the antiviral response of the body. The viral mechanisms associated with IFN-I evasion are multifaceted, including sequestering and shielding RNA within double-membrane vesicles, modification of viral mRNA 5-cap structures, and specific targeting of antiviral cellular pathways. In SARS-CoV and MERS-CoV, IFN-I production is protective only at the early stages after infection; at later time points, on the contrary, when the immune response is increased, IFN-I and inflammatory cytokines become pathogenic^66–69^. In both zika virus and dengue virus, fenretinide inhibited the non-structural protein 5 (NS5), which contributed to virulence, by preventing the production of IFN-I^70^. Thus, it is possible that fenretinide may also influence the mechanisms regulating IFN-I evasion in coronavirus infections. However, there are still few relevant findings, and the precise mechanism of action is unclear, calling for more in-depth pharmacological study.

Our literature search found a lack of research on the shared mechanism between COVID-19 and PH, especially bioinformatic studies. Here, we screened for C-DEGs, tested Core genes with a machine algorithm, and built a model to predict the COVID-19 combined with PH. We investigated how these two essential genes are linked to diseases and made predictions about the transcription factors and miRNAs that regulate them. In the end, we used molecular docking and targeting predictions for two important genes to determine which medications would be most effective.

However, there were certain gaps in our research. First, through molecular docking, we found that Fenretinide, a targeted drug for SELE and CCL20, may be a new target for the treatment of COVID 19 combined PH, though the mechanism by which it acts still needs to be further studied. External validation is needed for additional verification of the current outcomes. Moreover, in vitro model validation is required to additionally explore the core gene functions.

## Materials and methods

### Data collection

We scoured the whole GEO database (http://www.ncbi.nlm.nih.gov/geo/) using the phrases "COVID-19" and "Pulmonary hypertension"^71^. All sequenced data came from humans, and all screened data sets included both control and diseased subjects. The GSE113439, GSE147507, and GSE53408 datasets passed a rigorous screening process and were ultimately chosen. There was a total of 23 patients with COVID-19 and 55 healthy volunteers' samples in the GSE147507, whereas the GSE113439 included 15 patients with PH and 11 normal controls. Furthermore, there were 11 healthy subjects and 12 with very severe PH in the GSE53408 dataset, 9 healthy subjects and 26 with COVID-19 in the GSE196822 dataset. An overview of these datasets is shown in Table S8. The genomic profile of DEGs was log2 transformed, and gene symbols were matched to probes using annotation documentation from relevant datasets. Eventually, a gene matrix was extracted for further analysis, with gene symbols being contained in columns and sample names represented in rows.

### DEGs screening

DEGs were identified between PH and controls in the GSE113439 dataset using the Limma and GEOquery packages in the R programme, and between COVID-19 cases and controls within GSE147507. If a gene ID could not be assigned to a probe ID during the conversion process, the probe ID was not used. After merging many probe IDs into a single gene ID, the final calculation was determined as the median expression value. Adjusted p-values less than 0.05 and |logFC (fold change) |≥ 1 were considered statistically significant.

### DEGs-related KEGG and GO analyses

We performed both KEGG and GO analyses to further comprehend the physiological functions and functional correlations of shared COVID-19 and PH DEGs. Adjusted p-values of < 0.05 were deemed significant. The top 10 DEGs were shown using R's clusterProfiler tool.

### C-DEGs-related enrichment and identification analyses

Using the VennDiagram tool (http://jvenn.toulouse.inra.fr/app/example.html), we could visually represent C-DEGs (both low- and highly-expressed ones) from the GSE147507 and GSE113439. R's cluster-Profiler tool was employed for displaying the results of the KEGG and GO network studies. Significance was defined as before. Three different machine-learning algorithms—support vector machine-recursive feature elimination (SVM-RFE), least absolute shrinkage and selection operator (LASSO) logistic regression, and random forests (RF)^72–77^—were utilized for identifying potential new biomarkers for pediatric sepsis. Additionally, the random forest method was implemented using the "randomForest" R package in R. Using the "glmnet" R package^78^, this study conducted LASSO logistic regression analysis, with minimum lambda being deemed best. Partial likelihood deviation was below 5%, and parameter selection for optimization was cross-verified by a factor of 10. The genes that share characteristics with all three of the earlier-discussed classification schemes were then chosen for further investigation.

### Nomogram construction and receiver operating characteristics curve (ROC)

Candidates for biomarkers had their levels of expression evaluated between the PH and Control groups so that the diagnostic value of each could be calculated using a receiver operating characteristic (ROC) curve. The diagnostic value was then estimated using a 95% confidence interval and the area under the ROC curve (AUC). As a means of reducing bias, the GSE53408 dataset was utilized for validation. For the rms R package nomogram construction, only those candidates with an AUC > 0.7 in both test and validation sets were chosen. AUC was used to verify the nomogram's diagnostic accuracy, and AUC and decision and calibration curves were used to evaluate the nomogram's performance.

### Immune invasion and GSEA analysis

Next, GSEA in R was utilized for evaluating the hub genes that could be detected. A phenotype's significance might be determined via evaluating the gene distribution pattern for that trait against a predefined gene set. Besides, to measure the degree of immune infiltration in each dataset sample, the ssGSEA score was employed for quantifying immune cell invasion in COVID-19 and PH datasets and for immune invasion establishment within GSE113439 and GSE147507. Correlations between 2 core genes and 23 immune cells were determined using Pearson correlation analysis to reveal immune cells and core genes potential correlations^79^.

### TF-gene and TF-miRNA modulatory networks

The Networkanalyst platform (www.networkanalyst.ca) has been utilized for creating TF-miRNA and TF-gene modulatory networks^80^. We then validate TF-gene and TF-miRNA network by FFL loop. FFLtool is available on http://bioinfo.life.hust.edu.cn/FFLtool/^81^.

### Molecular docking simulations and evaluation of candidate drugs

Understanding protein activities and furthering drug development are both aided by chemical-protein interaction networks. Analysis was done using the web-based Enrichr portal together with the Drug Signatures Database (DSigDB, http://dsigdb.tanlab.org/DSigDBv1.0/^82^. Based on their p-values, the top 8 therapeutic compounds were selected as potential candidates. The Protein Data Bank (www.rcsb.org) served as the source for the crystal compositions of these important proteins, and Autodock tools (version 1.5.4) were approached in all docking studies. Binding energy was approached to demonstrate the findings. The ultimate outcome was displayed in Pymol (PyMOL Molecular Visualisation System 2020). 2D interaction analysis and statistics of interaction types, distances, and numbers were performed using the academic version of Maestro software (https://newsite.schrodinger.com/platform/products/maestro/).

### Molecular dynamics (MD) simulations

GROMACS 2022.3 software was used for molecular dynamics simulations. For small-molecule preprocessing, AmberTools 22 was used to add GAFF force fields to small molecules, while Gaussian 16 W was used to hydrogenate small molecules and calculate the RESP potential. Potential data were added to the topology file of the molecular dynamics system. The simulation conditions were carried out at a static temperature of 300 K and an atmospheric pressure of 1 Bar. Amber99sb-ildn was used for force fields, water molecules were used as the solvent (Tip3p water model), and the total charge of the simulation system was neutralized by adding an appropriate number of Na^+^ ions. The simulation system adopted the steepest descent method to minimize the energy, and then conducted the isothermal isovolumic ensemble (NVT) equilibrium and isothermal isobaric ensemble (NPT) equilibrium for 100,000 steps, respectively, with a coupling constant of 0.1 ps and a duration of 100 ps. The free molecular dynamics simulation was then performed. The process consisted of 5,000,000 steps, with a step length of 2 fs and a total duration of 100 ns. After completion of the simulation, the built-in tool of the software was used to analyze the trajectory, and the root mean-square deviation (RMSD), root-mean-square fluctuation (RMSF), and protein rotation radius of each amino acid trajectory were calculated, combined with the free energy (MMPBSA), free energy topography, and other data^83–85^.

### Statistical analysis

T-tests were used to compare the proportions of different immune cells between the control and PH groups using GraphPad Prism Version 8.3.0 (GraphPad Software, San Diego, CA, USA). P-values < 0.05 were considered statistically significant.

## Conclusions

In this research, SELE and CCL20 were found to be indicators of COVID-19 and PH co-pathogenesis by various bioinformatics analyse and machine learning algorithms. Adaptive immune response, leukocyte, lymphocyte mediated immune responses, and proinflammatory response mediated by cytokines like IL-12, TNF-a were functionally enriched in these two hub genes. These two hub genes were selected for nomogram construction and their diagnostic value evaluated by machine learning. The nomogram was found to have high diagnostic value. Dendritic cells had the strongest connection with SELE and CCL20, followed by activated CD4 T cells, active dendritic cells, natural killer T cells, neutrophils, and plasmacytoid dendritic cells. Using only 2 reference genes, we were able to isolate 12 shared TFs and 25 shared TF-miRNAs.by FFL tool, This FFL among CCL20, miR-1256 and PPARG may be a novel regulatory module in COVID-19 complicated with pulmonary hypertension. It was hypothesized that FENRETINIDE, 1-NITROPYRENE, and AFLATOXIN B1would be useful in treating COVID-19 complicated by PH. Further molecular docking and molecular dynamics simulations showed that 1-nitropyrene had the most stable binding with SELE and CCL20. Understanding the comorbidity of COVID-19 and PH may be aided by these biomarkers and the connection between COVID-19 and PH and angiogenesis.

### Supplementary Information

Supplementary Information 1.

Supplementary Information 2.

## Data Availability

Datasets used in the study (GSE113439, GSE147507,GSE53408 and GSE196822) can be downloaded without restriction from the public GEO database.GSE113439: https://www.ncbi.nlm.nih.gov/geo/query/acc.cgi? acc = GSE113439; GSE147507: https://www.ncbi.nlm.nih.gov/gds/? acc = GSE147507; GSE53408: https://www.ncbi.nlm.nih.gov/geo/query/acc.cgi? acc = GSE53408;.https://www.ncbi.nlm.nih.gov/geo/query/acc.cgi? acc = GSE196822;.The original contributions presented in the study are included in the article/Supplementary Material. Further inquiries can be directed to the corresponding author.

## Abbreviations

COVID: Coronavirus disease
PH: Pulmonary hypertension
GEO: GEne expression omnibus
DEGs: Differentially expressed genes
KEGG: Kyoto encyclopedia of genes and genomes
GO: Gene ontology
SVM-RFE: Support vector machine recursive feature elimination
LASSO: Least absolute shrinkage and selection operator
RF: Random forest
