# Supplementary figures and images for: Potential therapeutic targets for COVID-19 complicated with pulmonary hypertension: a bioinformatics and early validation study
Source: Sci Rep. 2024 Apr 23;14:9294. doi: 10.1038/s41598-024-60113-7 (PMC11039624; doi:10.1038/s41598-024-60113-7)

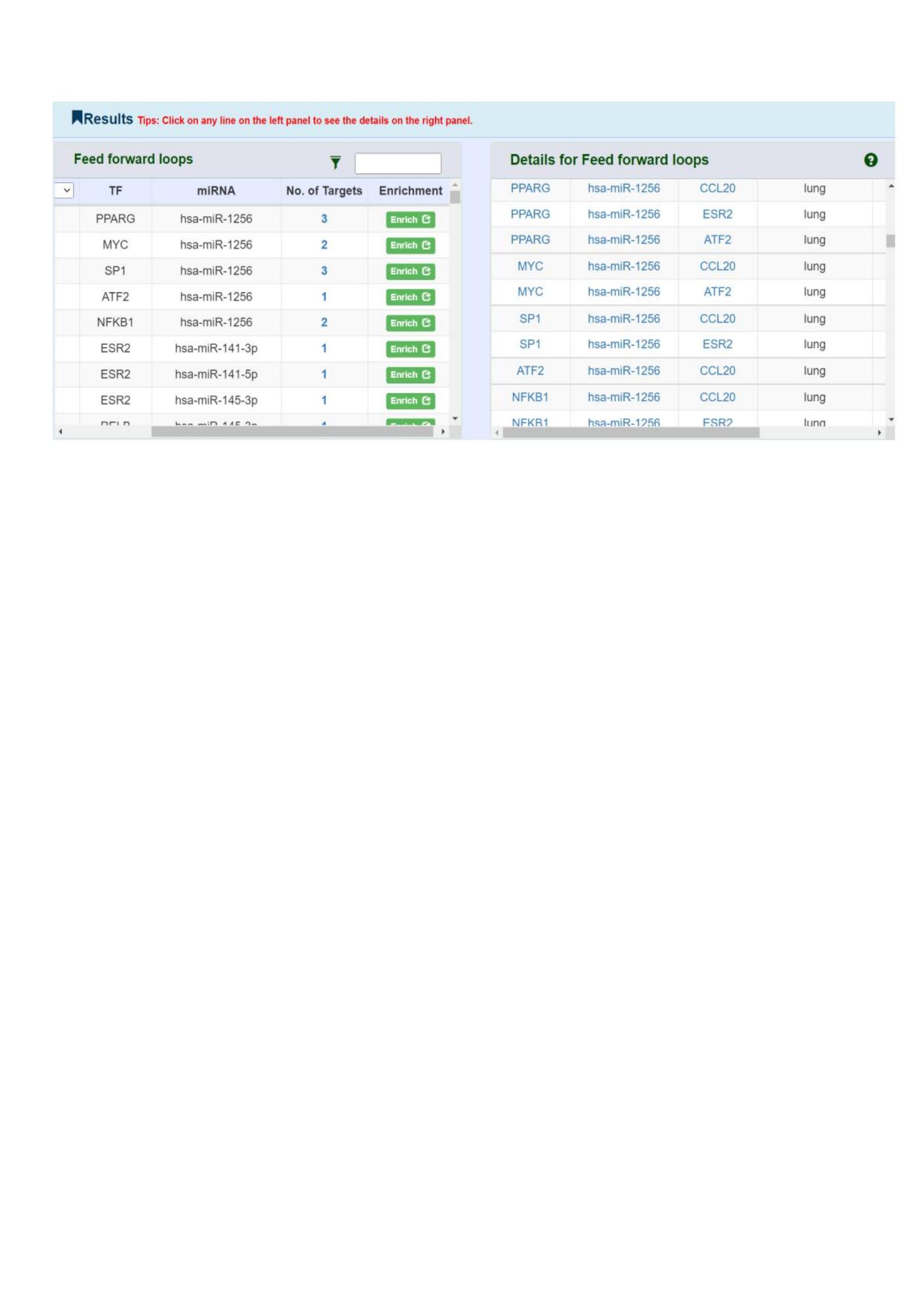

Supplement: Supplementary file 1 — Supplementary Information 1. [file 41598_2024_60113_MOESM1_ESM.jpg]
