# Supplementary material for: Potential therapeutic targets for COVID-19 complicated with pulmonary hypertension: a bioinformatics and early validation study
Source: Sci Rep. 2024 Apr 23;14:9294. doi: 10.1038/s41598-024-60113-7 (PMC11039624; doi:10.1038/s41598-024-60113-7)
Supplement: Supplementary file 2 — Supplementary Information 2. [file 41598_2024_60113_MOESM2_ESM.doc]

Supplementary Material

Potential therapeutic targets for COVID-19 complicated with pulmonary hypertension: a bioinformatics and early validation study

# Supplementary Tables

Table S1 Basic information for common DEGs

|  | Expression | GSE113439  Log2(FC) | GSE147507  Log2(FC) |
| --- | --- | --- | --- |
| GPR146 | Down | -1.444660667 | -1.158085697 |
| CLEC3B | Down | -1.100255939 | -7.183211624 |
| GGTLC1 | Down | -1.061782182 | -3.009524776 |
| PPP1R14A | Down | -1.048105758 | -1.025700054 |
| GDPD3 | Down | -1.269282545 | -1.169681427 |
| MAL | Down | -1.214943091 | -5.670535441 |
| CA4 | Down | -1.070570606 | -6.548971659 |
| MYOZ1 | Down | -1.503947697 | -2.888196638 |
| MS4A15 | Down | -1.503947697 | -5.239063833 |
| CCL5 | Down | -1.021894182 | -1.594261106 |
| NXF3 | Down | -1.042239879 | -3.471749244 |
| HIF3A | Down | -1.054738121 | -6.754072429 |
| VIPR1 | Down | -1.347693394 | -2.062863636 |
| FCER1A | Down | -1.047925212 | -2.962974622 |
| TMEM100 | Down | -1.339304364 | -3.474917228 |
| ACE2 | Up | 1.063780545 | 2.236035563 |
| ACSL1 | Up | 1.24898303 | 1.388430159 |
| ALDH1L2 | Up | 1.21050903 | 1.205722978 |
| ANKRD12 | Up | 1.027155394 | 1.337254858 |
| ANKRD22 | Up | 1.027155394 | 1.337254858 |
| AQP9 | Up | 1.159574606 | 6.958537534 |
| ARID4A | Up | 1.173162788 | 1.234059619 |
| BIRC3 | Up | 1.362014485 | 1.458890228 |
| BTAF1 | Up | 1.017496606 | 1.324035587 |
| CCL20 | Up | 1.270028848 | 1.901090342 |
| CEP350 | Up | 1.254649939 | 1.1552101 |
| CHD2 | Up | 1.144185394 | 2.030725258 |
| ESF1 | Up | 1.125811091 | 1.132860225 |
| FRK | Up | 1.091640788 | 1.155965881 |
| FRS2 | Up | 1.028519576 | 2.119658121 |
| GAPT | Up | 1.040946788 | 7.764567689 |
| HP | Up | 1.558253091 | 3.604375543 |
| IL18RAP | Up | 1.00180697 | 3.313682452 |
| IRAK3 | Up | 1.045325455 | 2.313574359 |
| MIER3 | Up | 1.03626303 | 1.270610487 |
| MMP8 | Up | 1.774119212 | 7.281504173 |
| MRC1 | Up | 1.171923333 | 1.974393803 |
| NBN | Up | 1.19304303 | 1.2035965 |
| NEXN | Up | 1.581548667 | 1.211714676 |
| NRIP1 | Up | 1.224161879 | 1.156002653 |
| NSRP1 | Up | 1.465172485 | 1.257760905 |
| PLIN2 | Up | 1.094728485 | 1.217433357 |
| PNN | Up | 1.034439333 | 1.77248262 |
| RB1CC1 | Up | 1.014354727 | 1.106853194 |
| RNF6 | Up | 1.161788485 | 1.111447932 |
| SAMD9L | Up | 1.252735758 | 2.237968495 |
| SAMSN1 | Up | 1.082200424 | 4.947930084 |
| SELE | Up | 1.358336667 | 2.241584284 |
| SLC7A2 | Up | 1.090394424 | 1.353329078 |
| SNORD20 | Up | 1.752948424 | 1.420382954 |
| SOCS4 | Up | 1.031108182 | 1.165278435 |
| SRFBP1 | Up | 1.318493273 | 1.249858267 |
| TFEC | Up | 1.240097939 | 5.806211741 |
| TIPARP | Up | 1.033065758 | 2.342877726 |
| TLR2 | Up | 1.003105091 | 1.534093007 |
| TNFAIP6 | Up | 1.020837697 | 2.899259181 |
| USP15 | Up | 1.054013394 | 1.401213661 |
| XRN1 | Up | 1.03695897 | 1.153242715 |
| ZNF267 | Up | 1.615219515 | 2.666322075 |
| ZNF292 | Up | 1.126315697 | 2.516628348 |
| ZNF654 | Up | 1.337059455 | 2.313206144 |
| ZNF845 | Up | 1.508093394 | 1.323153991 |

Table S2 KEGG enrichment analysis of common DEGs

| Category | Term | Count | Padj | Fold Enrichment |
| --- | --- | --- | --- | --- |
| KEGG_PATHWAY | TNF signaling pathway | 4 | 5.0×10^-4^ | 10.60776942 |
| KEGG_PATHWAY | Herpes simplex virus 1 infection | 6 | 5.7×10^-3^ | 3.542829241 |
| KEGG_PATHWAY | Rheumatoid arthritis | 3 | 3.4×10^-3^ | 9.752304147 |
| KEGG_PATHWAY | Viral protein interaction with cytokine and cytokine receptor | 3 | 4.2×10^-3^ | 9.069642857 |
| KEGG_PATHWAY | Malaria | 2 | 1.2×10^-2^ | 12.09285714 |
| KEGG_PATHWAY | Lipid and atherosclerosis | 3 | 3.3×10^-2^ | 4.218438538 |
| KEGG_PATHWAY | Inflammatory bowel disease | 2 | 1.9×10^-2^ | 9.302197802 |
| KEGG_PATHWAY | PPAR signaling pathway | 2 | 2.5×10^-2^ | 8.061904762 |
| KEGG_PATHWAY | mRNA surveillance pathway | 2 | 4.0×10^-2^ | 6.233431517 |

Table S3 GO enrichment analysis of common DEGs

| Category | Term | Count | Padj | Fold Enrichment |
| --- | --- | --- | --- | --- |
| GOTERM_BP_DIRECT | cellular response to interferon-gamma | 4 | 0.00023 | 13.29498374 |
| GOTERM_BP_DIRECT | inflammatory response | 6 | 0.0019 | 4.612862348 |
| GOTERM_BP_DIRECT | regulation of inflammatory response | 3 | 0.0038 | 9.584005266 |
| GOTERM_BP_DIRECT | neutrophil activation | 2 | 0.0012 | 38.71186441 |
| GOTERM_BP_DIRECT | positive regulation of T cell migration | 2 | 0.0015 | 34.63693131 |
| GOTERM_BP_DIRECT | immune response | 5 | 0.017 | 3.310370699 |
| GOTERM_BP_DIRECT | response to lipopolysaccharide | 3 | 0.0094 | 6.903164632 |
| GOTERM_BP_DIRECT | lipid storage | 2 | 0.0033 | 23.50363196 |
| GOTERM_BP_DIRECT | positive regulation of NF-kappaB transcription factor activity | 3 | 0.014 | 5.982742681 |
| GOTERM_BP_DIRECT | leukocyte cell-cell adhesion | 2 | 0.0042 | 20.56567797 |
| GOTERM_CC_DIRECT | membrane raft | 5 | 0.00073 | 7.014965986 |
| GOTERM_CC_DIRECT | tertiary granule lumen | 3 | 0.00055 | 18.74909091 |
| GOTERM_CC_DIRECT | specific granule lumen | 3 | 0.00078 | 16.63225806 |
| GOTERM_CC_DIRECT | plasma membrane | 24 | 0.011 | 1.546896681 |
| GOTERM_CC_DIRECT | nucleus | 24 | 0.048 | 1.367412564 |
| GOTERM_MF_DIRECT | transmembrane signaling receptor activity | 4 | 0.0025 | 7.071668533 |
| GOTERM_MF_DIRECT | threonine phosphatase inhibitor activity | 2 | 0.00068 | 51.13360324 |
| GOTERM_MF_DIRECT | cyclic ADP-ribose generating | 2 | 0.001 | 41.54605263 |
| GOTERM_MF_DIRECT | NAD(P)+ nucleosidase activity | 2 | 0.001 | 41.54605263 |
| GOTERM_MF_DIRECT | DNA binding | 9 | 0.025 | 2.105077966 |

TableS4 core genes in three independent computational methods

| RF | LASSO | SVM-RFE |
| --- | --- | --- |
| FRS2 | CLEC3B | CCL20 |
| CCL20 | MAL | NEXN |
| SELE | CA4 | TIPARP |
| ALDH1L2 | MYOZ1 | SELE |
| CEP350 | CCL5 | CCL5 |
| ZNF267 | NXF3 | TFEC |
| SLC7A2 | ACE2 | ZNF654 |
| ARID4A | ALDH1L2 | VIPR1 |
| ZNF292 | ANKRD22 |  |
| NRIP1 | BIRC3 |  |
|  | CCL20 |  |
|  | CHD2 |  |
|  | NEXN |  |
|  | PLIN2 |  |
|  | SELE |  |
|  | SLC7A2 |  |
|  | SNORD20 |  |
|  | TFEC |  |
|  | ZNF654 |  |
|  | ZNF845 |  |

TableS5 Candidate drugs (top eight) identified from gene–drug interaction enrichment analysis

| Name | Ajusted P-value |
| --- | --- |
| glutathione CTD 00006035 | 4.59×10^-05^ |
| Simvastatin and niacin BOSS | 8.97×10^-05^ |
| FENRETINIDE CTD 00007166 | 1.29×10^-04^ |
| 1-NITROPYRENE CTD 00001569 | 1.36×10^-04^ |
| N-Acetyl-L-cysteine CTD 00005305 | 2.47×10^-04^ |
| vincristine CTD 00006988 | 0.003872087 |
| hydrogen peroxide CTD 00006118 | 0.017856395 |
| AFLATOXIN B1 CTD 00007128 | 0.023724728 |

TableS6 The binding sites and energies for key drug targets were evaluated through AutoDock calculations

| Drug targets | Binding energy |
| --- | --- |
| **SELE** |  |
| glutathione CTD 00006035 | -4.6 |
| Simvastatin and niacin BOSS | -5.8 |
| FENRETINIDE CTD 00007166 | -7 |
| 1-NITROPYRENE CTD 00001569 | -6.8 |
| N-Acetyl-L-cysteine CTD 00005305 | -4.4 |
| vincristine CTD 00006988 | -6.3 |
| hydrogen peroxide CTD 00006118 | -2.7 |
| AFLATOXIN B1 CTD 00007128 | -7 |
| **CCL20** |  |
| glutathione CTD 00006035 | -5.3 |
| Simvastatin and niacin BOSS | -8.5 |
| FENRETINIDE CTD 00007166 | -8.8 |
| 1-NITROPYRENE CTD 00001569 | -9.2 |
| N-Acetyl-L-cysteine CTD 00005305 | -4.5 |
| vincristine CTD 00006988 | -8.3 |
| hydrogen peroxide CTD 00006118 | -2.7 |
| AFLATOXIN B1 CTD 00007128 | -8.9 |

Table S7: Potential Therapeutic Drugs Identified from the CTD Database for COVID-19 and PH

|  | SELE | | CCL20 | |
| --- | --- | --- | --- | --- |
| Interacting Chemical | References | Organisms | References | Organisms |
| Lipopolysaccharides | 5 | 2 | 10 | 2 |
| bisphenol A | 3 | 1 | 3 | 3 |
| Acetaminophen | 2 | 2 | 2 | 2 |
| Benzo(a)pyrene | 2 | 2 | 6 | 1 |
| Silicon Dioxide | 2 | 2 | 7 | 3 |
| Tetrachlorodibenzodioxin | 2 | 2 | 6 | 2 |
| titanium dioxide | 2 | 2 | 4 | 2 |
| 1-nitropyrene | 1 | 1 | 3 | 1 |
| 2,2',4,4'-tetrabromodiphenyl ether | 1 | 1 | 1 | 1 |
| 2-anisidine | 1 | 1 | 1 | 1 |

Table S8 Summary of those three GEO datasets

| ID | GSE number | Platform | Samples | Source types | Disease |
| --- | --- | --- | --- | --- | --- |
| 1 | GSE113439 | GPL7215 | 15 patients and 11 normal controls | Lung sample | PH |
| 2 | GSE147507 | GPL18573 | 23 patients and 55 normal controls | Lung sample | COVID-19 |
| 3 | GSE53408 | GPL6244 | 15 patients and 11 normal controls | Lung sample | PH |
| 4 | GSE196822 | GPL20301 | 26 patients and 9 healthy subjects | Whole blood | COVID-19 |
